# Primary treatment of type B post-axial ulnar polydactyly: A systematic review and meta-analysis

**DOI:** 10.1016/j.jpra.2022.05.002

**Published:** 2022-05-13

**Authors:** Harsh Samarendra, Ryckie G. Wade, Louise Glanvill, Justin Wormald, Abhilash Jain

**Affiliations:** a Department of Plastic and Reconstructive Surgery, Imperial College Healthcare NHS Trust, Flat 2, 17 Wyfold Road, London SW6 6SE, UK; b Department of Plastic and Reconstructive Surgery, Leeds Teaching Hospitals Trust, Leeds, UK; c Faculty of Medicine and Health Sciences, University of Leeds, Leeds, UK; d Department of Plastic and Reconstructive Surgery, Oxford University Hospitals NHS Foundation Trust, UK; e Department of Plastic and Reconstructive Surgery, Buckinghamshire Healthcare NHS Trust, Aylesbury, UK; f Nuffield Department of Orthopaedics, Rheumatology and Musculoskeletal Sciences, University of Oxford, Oxford, UK

**Keywords:** Ulnar polydactyly, Adverse events, Patient-reported outcomes, Surgical outcomes, Excision ligation

## Abstract

Optimal management of pedunculated ulnar polydactyly is not defined. This systematic review summarises objective and patient-reported outcomes following primary treatment. Two authors screened articles for inclusion according to a PROSPERO published protocol. The meta-analysis of adverse events was performed, and a narrative synthesis of satisfaction and patient-reported outcomes was reported. The risk of bias was assessed using Cochrane's ROBINS-I tool. Of 1650 articles identified, 15 were eligible, including 13 single-arm and 2 multi-arm studies. Complications were 6 times as likely with ligation procedures (22%), compared to surgical removal (1%) whether this was performed in the outpatient setting or operating theatre (OR 6.89 [95% CI 1.73, 27]). Parent-reported satisfaction was high for all treatments. Studies were at high risk of bias and low methodological quality. Outcome measurement and follow-up were heterogenous. Well-designed prospective observational and experimental studies are required to inform practice, incorporating clinician and parent-reported outcomes and economic analyses.

Level of evidence: I.

## Introduction

Ulnar (post-axial) polydactyly is a congenital upper limb anomaly characterised by the presence of an extra digit on the ulnar border of the hand at birth. Two distinct subtypes are recognised (Temtamy and MsKusick): type A, in which a fully developed extra digit articulates with either the fifth metacarpal or a duplicated metacarpal; or more commonly type B, a rudimentary non-functional digit that is attached by a soft tissue bridge.^1^ Approximately three quarters of cases of type B ulnar polydactyly are bilateral, and 85% have a family history.^1^^,^^2^

The treatment of type B ulnar polydactyly is the removal of the non-functioning accessory digit, which carries a mild degree of functional disability, to restore a normal appearance of the hand.^3^^,^^4^ The most effective treatment remains a topic of debate, and there is substantial variation in practice globally.^4^ Two treatments are most frequently reported: (1) suture/clip ligation^2^ or (2) surgical excision.^5^^,^^6^

Ligation involves tying off the base of the skin bridge with (typically) either a suture or metal clip, thereby inducing ischaemic necrosis and auto-amputation of the accessory digit. It is regarded as effective, well tolerated by patients and families and may avoid costs and complications associated with hospital admission and surgery under anaesthesia.^2^^,^^7^ The disadvantages include a delay to autoamputation, a residual skin bridge that may be problematic in later life, risk of treatment failure and infection.^2^^,^^4^

Surgical excision of the accessory digit may be performed in the outpatient clinic or operating theatre, under local or general anaesthesia depending on the age of the infant. Surgery may facilitate more proximal excision of accessory nerves, therefore, theoretically reducing the incidence of neuroma^2^^,^^8^ and may improve the aesthetic outcome.^9^^,^^10^ Potential disadvantages include parental distress whilst awaiting treatment, need for general anaesthesia in older infants, and increased costs associated with hospital admission and an operative procedure.^11^ One systematic review reports that complications are more prevalent following ligation compared with surgical excision; however, significant methodological flaws render their results unreliable.^4^

The aim of this systematic review is to compare the outcomes of suture ligation, office-based excision and operating room-based excision in type B ulnar polydactyly through a systematic appraisal of the literature.

## Methods

The study was conducted in accordance with the Cochrane handbook for systematic reviews of interventions and was reported in accordance with the Preferred Reporting Items for Systematic Reviews and Meta-Analyses (PRISMA) guidelines. The protocol was registered on Prospero (CRD42019156164).

### Search strategy

The following databases were searched through the OVID search platform: (1) PubMed, (2) EMBASE, (3) PsycINFO, and (4) the Cochrane Library on October 30, 2019 to identify all articles reporting outcomes in primary interventions for type B ulnar polydactyly. See Supplementary Figure 1 for the search strategy in full.

### Study selection

Studies that assessed outcomes following primary intervention for type B ulnar polydactyly were determined as eligible for inclusion. We included observational and experimental studies of any design. Case reports, review articles with no new primary data, and commentaries were excluded. Titles and abstracts were reviewed by two independent reviewers (LG and HS) against the inclusion and exclusion criteria, and discrepancies were resolved through discussion with a third independent reviewer (JW). The full texts of those considered to be potentially eligible for inclusion were reviewed by two independent reviewers against the inclusion/exclusion criteria. Any disagreements were resolved by discussion and review by a third independent reviewer.

### Data extraction

Interventions have been grouped into the following three treatment methods: (1) Suture/clip ligation, (2) office based/minor treatment room excision and (3) operating room-based excision (under either local and general anaesthesia). A pre-piloted proforma was used to extract data from the studies that are included for analysis. The extracted information includes baseline characteristics, participant demographics and details of the intervention methods and study design. The outcomes for synthesis were as follows: (1) the prevalence of adverse events (clinician or parent-reported outcomes); (2) patient/parent satisfaction or ratings of appearance; (3) other clinician-assessed postoperative outcomes. Additional outcomes of interest include parent expressions of preference or motivation in treatment, local pathways, and influence of family history on decision-making.

### Risk of bias in individual studies

The risk of bias was assessed using the risk of bias In Non-randomised Studies of Interventions (ROBINS-I) tool and presented using Robvis.^12^ Studies were assessed by two independent reviewers, and disagreements were resolved by discussion (HS/LG).

### Synthesis of results

The pooled prevalence of complications was estimated using the *metaprop* package in Stata/MP v15. DerSimonian and Laird random effects were used given the clinical heterogeneity. The Freeman-Tukey arcsine transformation was used to stabilise the variance. However, 95% confidence intervals (CIs) around the study-specific and pooled prevalence were computed using the score-test statistic. Variations in the prevalence of complications were explored by meta-regression using *metareg*.^13^ P-values were permuted with 20,000 iterations. Statistical heterogeneity was assessed by I^2^. A z-test (and the corresponding *p*-values) assessed whether the observed prevalence was different from zero per cent. To assess possible small-study effects (or publication bias across studies), we produced a funnel plot using *metafunnel*.

## Results

### Study selection

**Figure 1:**
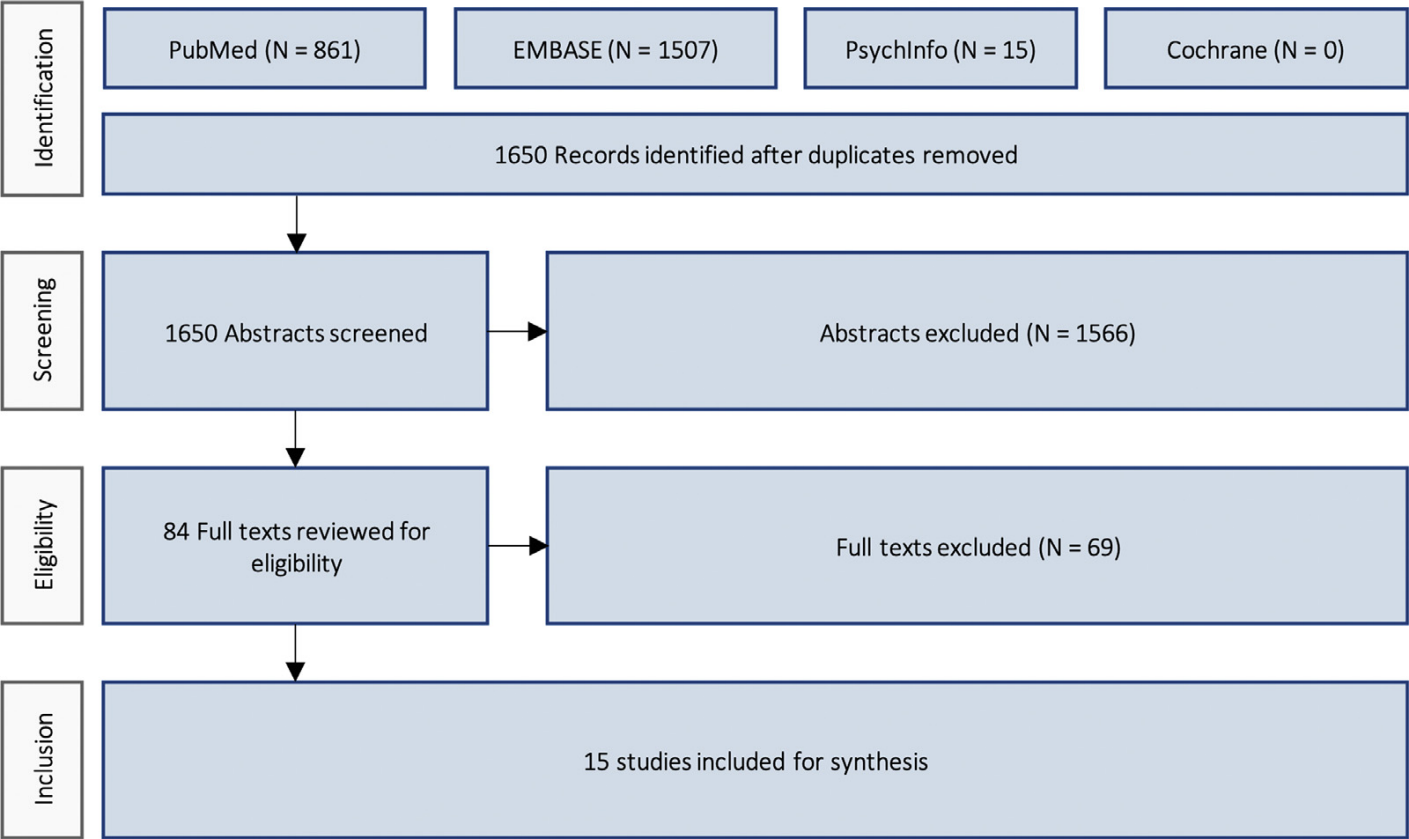
PRISMA flow diagram.

After screening 1650 records, 15 studies^2^^,^^5^^,^^16^, ^17^, ^18^, ^19^, ^20^^,^^6^, ^7^, ^8^, ^9^, ^10^, ^11^^,^^14^^,^^15^ were included (PRISMA flowchart, as shown Figure 1).

### Study characteristics

The studies originated from different 5 countries (UK, USA, Israel, India and Austria; Table 1). All were cohort studies, three prospective^2^^,^^1^^,^^20^ and 12 retrospective.^5^^,^^6^^,^^18^^,^^19^^,^^7^, ^8^, ^9^, ^10^^,^^14^, ^15^, ^16^, ^17^ There were two multi-arm studies^8^^,^^11^ whilst the remainder was single-arm studies. Across all studies, interventions to remove the accessory digit were performed from 2 days of age^20^ up to a maximum of 4.2 years^6^ with an average age of 96 days. Revisional procedures were performed up to a maximum age of 13 years.^9^ Age of intervention varied with treatments, with ligation performed from 1 week^2^ to 40 weeks of age,^7^ clinic-based excision from 2 days^20^ to 4.2 years^6^ and OR-based excision from 3 days^18^ to 36 months.^19^ Where reported, the male/female ratio was 1.3:1 (Table 2).

**Table 1:** Study design and characteristics.

Author (date)	Location	Study design	Participants	Study size	Digits	Age at intervention	M:F ratio	Previous intervention	Procedure
Ligation									
Watson & Henrikus (1997)	USA	Prospective case series	Type B (T&M)	21	37	(1–2 weeks)	12:9	None	Suture ligation in neonatal nursery
Mills (2014)	USA	Retrospective case series	Type B (T&M) base of digit < 6mm	132	231	8 weeks (2–40 weeks)	77:55	None	OPD clip application with LA
Clinic-based excision									
Katz (2011)	Israel	Prospective case series	Type B (T&M)	11	15	(2–3 days)	6:5	None	LA excision, neonatal minor ops room
Carpenter (2015)	USA	Retrospective case series	Type B (T&M)	26	38	3.3months (9 days - 4.2 years)	NR	5 patients had previous suture ligation, now being treated for residual bump	LA excision, office-based
Al Hassani (2019)	UK	Retrospective case series	Stelling I	28	43	69 Days (27–134)	14:16	None	LA excision in paediatric minor treatment unit
Operating theatre-based excision									
Stewart (2001)	UK	Retrospective case series	‘Minor forms’ of U.P.	40	NR	3 days (1–14 days)	NR	None	LA surgical excision
Leber (2003)	USA	Retrospective case series	Pedunculated supernumerary digits	6	11	(3 weeks – 21 months)	3:3	none	Surgical excision
Mullick (2010)	USA	Retrospective case series	Type B (T&M)	10	13	(1 week – 13 years)	5:5	All presented with incomplete amputations, painful neuroma or both – prev. ligation	GA surgical excision
Khan (2012)	India	Retrospective case series	Stelling IIa UP	10	20	18.2 months (6–36 months)	5:5	None	GA surgical excision
Ahmad (2014)	UK	Retrospective case series	Type B (T&M)	86	135	(<10 weeks)	NR	None	LA surgical excision
Singer (2014)	Austria	Retrospective case series	Stelling I/II/III	32	41	8.6 months (0–10months)	NR	NR	GA surgical excision
Macdonald (2017)	UK	Retrospective case series	Type B (T&M)	20	NR	(< 6 weeks)	NR	None	LA surgical excision
Shirley (2019)	UK	Retrospective case series	Stelling I	40	58	(3 – 18 weeks)	NR	None	LA surgical excision
Comparative studies									
Rayan (2000)	USA	Retrospective cohort study	All types UP (82% Stelling I/II)	122	123	NR	85:63	None	Suture ligation Surgical excision No treatment
Samra (2016)	USA	Prospective cohort study	Type B (T&M)	14	25	NR	NR	None	Suture ligation LA excision in clinic, GA excision in OR

**Table 2:** Adverse events and patient-reported outcomes.

Author	Admission (Y/N/NR)	FU period	Overall complication	Treatment failure	Neuroma	Nubbin Unaesthetic scar	Revision procedure	Parent reported outcomes
Ligation								
Watson & Henrikus (1997)	N	1–2 weeks, then 15/21 patients at 20 months	57.1%	4.7%	NR	52.4%	4.7%	No parent reported outcomes
Rayan (2000) LIGATION *N* = 105	NR	1–2 weeks (some at 12–37 months)	23.5%	NR	6.7%	16.1%	2.8%	No parent reported outcomes
Mills (2014)	NR	10–14 days	10.7%	NR	7%	3.7%	7%	No parent reported outcomes
Samra (2016) LIGATION *N* = 2	NR	1 month and 3 months	0%	NR	NR	NR	NR	0 – 10 satisfaction score 10 Higher perceived pain 5/10
Clinic-based excision								
Katz (2011)	NR	Post-op Day 1, 1 week, 1 year	0%	0%	NR	0%	0%	‘All expressed satisfaction with cosmetic result’
Carpenter (2015)	N	NR	7.7%	NR	NR	NR	NR	No parent reported outcomes
Samra (2016) LA Excision *N* = 10	NR	1 month and 3 months	0%	NR	NR	NR	NR	9.8/10 satisfaction score
Al Hassani (2019)	N	Telephone questionnaire	NR	NR	NR	NR	NR	100% rated scar as excellent. 24 would recommend, 4 would not
Operating theatre-based excision								
Rayan (2000) EXCISION *N* = 27	NR	1–2 weeks (some at 12–37 months)	3.7%	NR	3.7%	0%	0%	No parent reported outcomes
Stewart (2001)	NR	None	NR	NR	NR	NR	NR	High levels of satisfaction 39/40 felt that scar barely noticeable
Leber (2003)	NR	Up to 6 years	0%	NR	NR	NR	0	All were pleased with cosmetic result
Mullick (2010)	NR	1–3 months post-op	0	0	0	0	0	Cosmetic outcome - All regarded ‘Acceptable’ to parents + surgeon
Khan (2012)	NR	2 years	10% (infection)	0	0	0	0	No parent reported outcomes
Ahmad (2014)	NR	None	2.2%	0%	0%	1.1%	0%	’Parents of patients were very pleased by the overall service’
Singer (2014)	NR	Questionnaire FU years later	10%	0	(5%) of nubbins	10%	NR	VAS 1–100 for function – score 89/100
SAMRA (2016) GA Excision *N* = 2	NR	1 month and 3 months1 month and 3 months	0%	NR	NR	NR	NR	10/10 satisfaction score Both did describe emotional distress (6/10) with having to wait
Macdonald (2017)	N	Telephone FU, 20 received formal survey	NR	NR	NR	NR	NR	19/20 very high overall satisfaction. 18/20 for scar
Shirley (2019)	N	Telephone questionnaire	5%	NR	NR	5%	NR	No parent reported outcomes

### Risk of bias assessment

**Figure 2:**
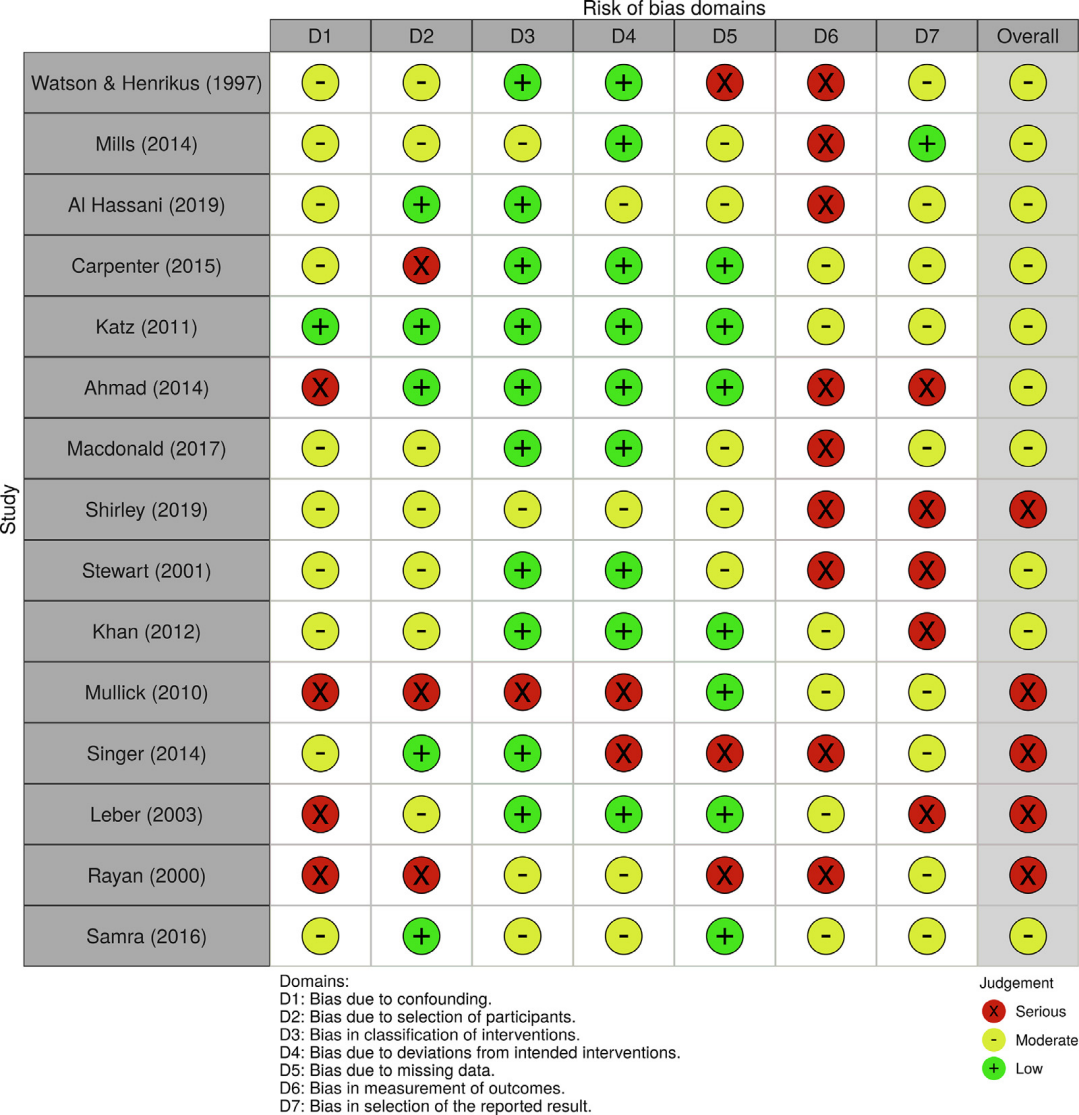
The summary risk of bias plot for included studies. Red = high risk; yellow = unclear risk; green = low risk.

The risk of bias was high across included studies. Common limitations include non-consecutive inclusion of patients, incomplete or selective follow-up (with 6 studies not providing follow-up at all) and a lack of systematic analysis of outcomes. A total of 13 out of 15 included studies are case series, in which bias is inherent in participant selection, absence of comparators and retrospective reviews of notes. A more detailed breakdown of risk as determined by the ROBINS-I tool is provided in Figure 2.

### Adverse events

**Figure 3:**
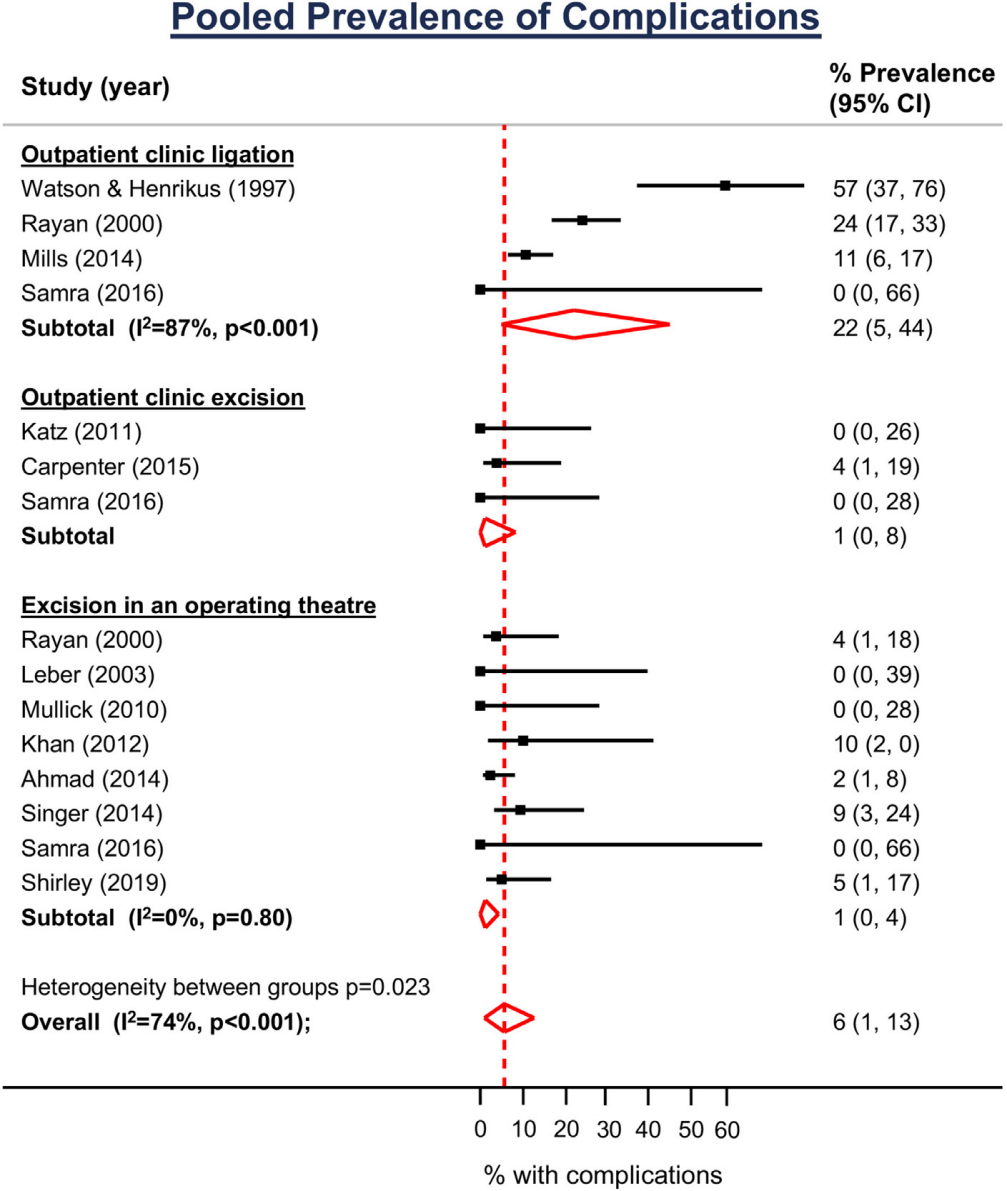
Forest plot showing the prevalence (%) of complications and 95% CIs. Estimates by pooled according to the method used to remove the accessory digit.

The pooled prevalence of complications was 6.0% (95% CI 1, 13; Figure 3). Complications were more common following ligation procedures (22.0% [95% CI 5, 44]; I^2^ 87%) compared to surgical excision in the outpatient setting (1.0% [95% CI 0, 8]) or operating theatre (1.0% [95% CI 0, 4] I^2^ 0%), although there was significant heterogeneity overall (I^2^ 74%).

Meta-regression showed that ligation was associated with 6 times the odds of complications (OR 6.89 [95% CI 1.73, 27] *p* = 0.013; permuted *p* = 0.012) when compared with surgical excision in either a theatre or the outpatient setting. Performing the procedure in an operating theatre did not reduce the risk of complications as compared to the outpatient clinic (OR 3.75 [95% CI 0.66, 21] *p* = 0.114).

**Figure 4:**
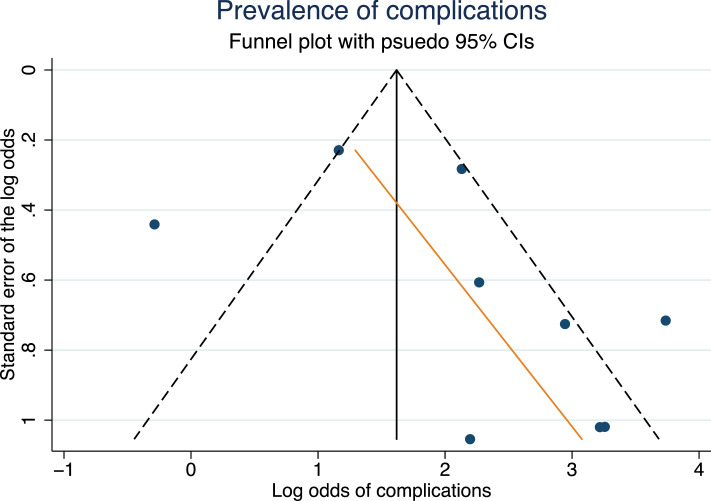
Funnel plot suggesting the presence of small-study effects (publication bias).

A funnel plot of the risk of complications against study precision showed that datapoints were strongly asymmetrical (Figure 4; Egger's regression co-efficient 0.80 [95% CI −0.69, 2.29], *p* = 0.246; Figure 4), which suggests the presence of small-study effects (publication bias).

### Patient (parent)-reported measures

There was insufficient data for a meta-analysis of patient-reported outcomes. Such data were available in one multi-arm study^11^ and four single-arm studies, and none were recognised validated measures. Samra et al. report no difference in overall satisfaction between ligation and excision groups; however, they note that infants’ perceived pain scores were higher in the suture ligation group, whilst emotional distress was higher for parents choosing excision under GA, which they attributed to the delay to intervention.^11^ Parental ratings of scar/cosmesis were assessed in 4 single-arm studies, and the scores were consistently high.^5^^,^^6^^,^^14^^,^^18^

## Discussion

This systematic review and metanalysis demonstrate a paucity of high-quality studies. The available evidence suggests that suture ligation carries a clinically and statistically significant higher risk of complications than surgical excision. Studies to date suggest a higher risk of aesthetically unacceptable remnant nubbins with suture/clip ligation, compared to excision^2^^,^^8^; however, the reliability of this observation is questionable due to a general lack of standardised outcome measurement and follow-up across the included studies. For example, between 2.8 and 7.0% of parents requested revisional procedures for remnant nubbins, suggesting they may not be as important to parents and patients as surgeons believe.^2^^,^^7^^,^^8^ A digital neuroma is infrequently assessed, though one study suggests a higher incidence after suture ligation (6.7%), compared with excision (3.7%)^8^; however, there is insufficient data on rates of revisional surgery.

Clinic-based and operating theatre-based excision should be recognised as distinct modalities owing to procedural differences and, in some cases, implications for anaesthesia and admission. This review finds comparable rates of adverse outcomes which suggests that some of the purported disadvantages of surgical excision that relate to treatment delay, distress due to perceived stigma and concern relating to general anaesthesia may be circumvented through clinic-based excision.^11^^,^^17^ The approach is best tolerated in younger infants, and well-established pathways for referral are key to timely delivery.^14^ Theatre-based local anaesthetic excision shares these advantages but comes with greater cost and resource consumption for services.

Our review shows a paucity of the patient and parent voice in current research, with only 5 studies evaluating this using archaic instruments. A postoperative questionnaire delivered by Samra et al.^11^ showed that parental satisfaction was high in all three treatment arms, despite differences in specific measures such as pain scores and satisfaction with cosmetic appearance. Some studies suggest that the timing of treatment is of paramount importance to parents. Shirley et al. commented that parents often express dissatisfaction with the stigma of accessory digits,^17^ and Stewart et al. ascribed the high satisfaction (39/40) rates in their study (LA excision at a median age of 3 days) to early treatment.^18^ Parent views on anaesthetic choice are also significant and not explored in the published literature.

Validated outcome measures may improve our assessment of both objective surgical outcomes, such as scar quality and patient/parent-reported outcomes, such as overall satisfaction with treatment and appearance.^21^ To ensure optimal impact and relevance to patients, future comparative studies should select patient- or parent-centred primary endpoints. Patient and public involvement is an integral to this aim, specifically, in devising pertinent research questions and selecting impactful study outcomes.^22^ Future study design should consider that, given the strong genetic component in type B ulnar polydactyly, parental perspective may in many cases be shaped by personal experience of treatment and complications.^10^^,^^15^

Future studies should also consider the cost and benefits of proposed treatments. Excision under general anaesthesia is associated with increased costs. One study estimates that excision incurs a 40-fold increase in costs when compared to office-based clip application.^7^ Office-based excision may also offer comparative savings to services. Al-Hassani et al. reported a decrease in procedure time from 59 min in the OR to 18 min.^14^ This finding is not consistent across studies, however, with one study reporting operating time for local anaesthetic treatment to be 19 min.^17^

The optimum management of type B ulnar polydactyly is unclear based on the available evidence. Furthermore, prior studies have not been designed with patient or public involvement. There is a pressing need for a prospective observational or experimental study assessing the benefits and drawbacks of different treatment options and their cost-efficacy in the modern health service, with all relevant stakeholders involved.

## Ethical approval declaration

Ethical approval was not sought for this review.

## CRediT authorship contribution statement

**Harsh Samarendra:** Conceptualization, Formal analysis, Data curation, Investigation, Software, Writing – review & editing. **Ryckie G. Wade:** Conceptualization, Formal analysis, Data curation, Investigation, Software, Writing – review & editing. **Louise Glanvill:** Conceptualization, Formal analysis, Data curation, Investigation, Software, Writing – review & editing. **Justin Wormald:** Conceptualization, Formal analysis, Writing – review & editing. **Abhilash Jain:** Conceptualization, Formal analysis, Writing – review & editing.

## Declaration of Competing Interest

The author(s) declare no potential competing of interest with respect to the research, authorship and/or publication of this article.

## Appendix Supplementary materials

Image, application 1
