# Supplementary material for: Primary treatment of type B post-axial ulnar polydactyly: A systematic review and meta-analysis
Source: JPRAS Open. 2022 May 13;34:21–33. doi: 10.1016/j.jpra.2022.05.002 (PMC9478869; doi:10.1016/j.jpra.2022.05.002)
Supplement: Supplementary file 1 [file mmc1.pdf]

Supplementary Figure 1 – Search strategy in full:

1 Polydactyly/
2 polydactyl*.ti,ab.
3 hyperdactyl*.ti,ab.
4 "supernumerary digit".ti,ab.
5 "accessory digit".ti,ab.
6 1 or 2 or 3 or 4 or 5
7 ligation/ or minor surgical procedures/
8 Postoperative Complications/
9 treatment outcome/ or treatment failure/
10 excision.ti,ab.
11 ligation.ti,ab.
12 complication*.ti,ab.
13 management.ti,ab.
14 treat*.ti,ab.
15 approach.ti,ab.
16 outcome*.ti,ab.
17 intervention.ti,ab.
18 7 or 8 or 9 or 10 or 11 or 12 or 13 or 14 or 15 or 16 or 17 (8136684)
19 6 and 18 (740)
20 19 (740)
